# Soil heterogeneity in the horizontal distribution of microplastics influences productivity and species composition of plant communities

**DOI:** 10.3389/fpls.2022.1075007

**Published:** 2022-12-08

**Authors:** Xiao-Mei Zhang, Xiao-Xiao Cao, Lin-Xuan He, Wei Xue, Jun-Qin Gao, Ning-Fei Lei, Jin-Song Chen, Fei-Hai Yu, Mai-He Li

**Affiliations:** ^1^ Institute of Wetland Ecology & Clone Ecology, Taizhou University, Taizhou, China; ^2^ College of Ecology and Environment, Chengdu University of Technology, Chengdu, China; ^3^ School of Ecology and Nature Conservation, Beijing Forestry University, Beijing, China; ^4^ College of Life Science, Sichuan Normal University, Chengdu, China; ^5^ Swiss Federal Institute for Forest, Snow and Landscape Research, Birmensdorf, Switzerland

**Keywords:** environmental heterogeneity, experimental plant communities, foraging response, microplastic heterogeneity, soil microplastics

## Abstract

Contamination of soils by microplastics can have profound ecological impacts on terrestrial ecosystems and has received increasing attention. However, few studies have considered the impacts of soil microplastics on plant communities and none has tested the impacts of spatial heterogeneity in the horizontal distribution of microplastics in the soil on plant communities. We grew experimental plant communities in soils with either a homogeneous or a heterogeneous distribution of each of six common microplastics, i.e., polystyrene foam (EPS), polyethylene fiber (PET), polyethylene bead (HDPE), polypropylene fiber (PP), polylactic bead (PLA) and polyamide bead (PA6). The heterogeneous treatment consisted of two soil patches without microplastics and two with a higher (0.2%) concentration of microplastics, and the homogeneous treatment consisted of four patches all with a lower (0.1%) concentration of microplastics. Thus, the total amounts of microplastics in the soils were exactly the same in the two treatments. Total and root biomass of the plant communities were significantly higher in the homogeneous than in the heterogeneous treatment when the microplastic was PET and PP, smaller when it was PLA, but not different when it was EPS, HDPE or PA6. In the heterogeneous treatment, total and root biomass were significantly smaller in the patches with than without microplastics when the microplastic was EPS, but greater when the microplastic was PET or PP. Additionally, in the heterogeneous treatment, root biomass was significantly smaller in the patches with than without microplastics when the microplastic was HDPE, and shoot biomass was also significantly smaller when the microplastic was EPS or PET. The heterogeneous distribution of EPS in the soil significantly decreased community evenness, but the heterogeneous distribution of PET increased it. We conclude that soil heterogeneity in the horizontal distribution of microplastics can influence productivity and species composition of plant communities, but such an effect varies depending on microplastic chemical composition (types) and morphology (shapes).

## Introduction

Pollution by microplastics is currently a serious environmental problem that receives increasing attention worldwide (Corradini et al., 2019; Roy et al., 2022; Zhang et al., 2022). Studies have shown that microplastics in soils can have profound impacts on survival, growth, morphology and physiology of individual plants (de Souza Machado et al., 2018a; Rillig et al., 2019; Pignattelli et al., 2020), likely *via* their effects on soil physico-chemical properties and soil microbial communities (de Souza Machado et al., 2018b; Wan et al., 2019). For instance, microplastics have been found to delay seed gemination (Bosker et al., 2019), reduce seed germination rate and seedling survival (Qi et al., 2018; Boots et al., 2019), modify tissue nutrient contents (de Souza Machado et al., 2019), alter root and shoot morphology (Boots et al., 2019; Rillig et al., 2019), and change biomass production and allocation (Cunha et al., 2020). A recent study has shown that microplastics in soils could also influence the productivity of plant communities and lead to changes in the dominant species within the communities (Lozano and Rillig, 2020).

The distribution of microplastics in soils in the horizontal space is often not uniform but heterogeneous (Rillig et al., 2017; Sun et al., 2022), i.e., microplastics are present in one soil microsite (patch) but absent in its horizontally adjacent soil microsites or microplastics are present in adjacent microsites with different concentrations. For instance, long-term plastic film shedding and random disposal of plastics may create soil patches with microplastics (Steinmetz et al., 2016; Blasing and Amelung, 2018; Zhang and Liu, 2018). Sewage sludge application, wastewater irrigation, human tillage, soil biota activity, atmospheric deposition and wind- or water-mediated movement may redistribute microplastics in soils and create horizontal soil patches with different concentrations of microplastics (Barnes et al., 2009; Nizzetto et al., 2016; Cai et al., 2017; Lwanga et al., 2017; Rillig et al., 2017). As microplastics in soils and their concentrations can influence plant growth (van Kleunen et al., 2020; Lozano et al., 2021; Li et al., 2022), soil heterogeneity in the horizontal distribution of microplastics may have significant ecological impacts on plant communities.

A large number of studies have assessed the ecological impacts of soil heterogeneity in the horizontal distribution of factors other than microplastics, including nutrients (Tsunoda et al., 2014; Xue et al., 2020; Adomako et al., 2021a; Gao et al., 2021), water (You et al., 2016; Wang et al., 2017), heavy metals (Roiloa and Retuerto, 2012; Xu and Zhou, 2017) and particle size of the soil (Baer et al., 2005; Huang et al., 2013; Xue et al., 2016). These studies have shown that soil heterogeneity in the distribution of such factors can affect growth, morphology and physiology of individual plants (Zhou et al., 2012; Tsunoda et al., 2014; Adomako et al., 2021b; Si et al., 2021), influence dynamics of plant populations (Hutchings et al., 2003; Baer et al., 2020), modify intraspecific and interspecific plant-plant interactions (Liang et al., 2020; Xue et al., 2021; Hu et al., 2022), and change plant community structure and ecosystem function (Wijesinghe et al., 2005; Yao et al., 2021). One underlying mechanism is that some plants can grow across patches and allocate more roots and/or shoots in favorable microsites (e.g., high-nutrient patches and patches not contaminated by heavy metals) and less in unfavorable microsites (e.g., low-nutrient patches and patches contaminated by heavy metals), showing foraging responses to increase resource harvesting (Hutchings et al., 2003; Tsunoda et al., 2014; Chen et al., 2019; Cao et al., 2022). Similarly, we hypothesize that soil heterogeneity in the horizontal distribution of microplastics can affect species composition and productivity of plant communities. So far, however, few studies have considered the impacts of soil microplastics on plant communities (Lozano and Rillig, 2020) and none has tested the impacts of soil heterogeneity in the horizontal distribution of microplastics on plant communities.

Microplastics are diverse in their types (chemical composition) and shapes (morphology) (Rillig et al., 2019; Li et al., 2022). Differences in the chemical composition and morphology of microplastics may result in differences in their impacts on soil physico-chemical properties and soil microbial communities (Huang et al., 2021). de Souza Machado et al. (2018b), for instance, have shown that polyester fibers increase water holding capacity, but polyacrylic fibers and polyethylene fragments have no significant effect. Also, microplastic fibers were found to have a larger impact on soil aggregation than other microplastic shapes (Lozano et al., 2021) and polyethylene foams increased soil pH more than polyethylene films (Zhao et al., 2021). Consequently, microplastics of different types and shapes can have different impacts on plant growth (Qi et al., 2018; de Souza Machado et al., 2019; Rillig et al., 2019). If microplastics in soils have a negative effect on plant growth due to their impacts on soil physico-chemical properties and soil microbial communities (de Souza Machado et al., 2019; Rillig et al., 2019; Li et al., 2022), then plants may allocate more shoots and/or roots in soil patches without microplastics than in their horizontally adjacent soil patches with microplastics. Such foraging responses may promote resource harvesting and thus increase the productivity of the whole plant communities. On the other hand, if microplastics in soils have no effect on plant growth, then the heterogeneous distribution of soil microplastics will not influence the species composition and productivity of plant communities. Therefore, we hypothesize that the impacts of soil heterogeneity in the horizontal distribution of microplastics on plant communities may vary depending on the chemical composition and morphology of microplastics.

To test these hypotheses, we grew experimental plant communities in soils with either a homogeneous or a heterogeneous distribution of each of six common microplastics. Specifically, we addressed the following questions: (1) Does spatial heterogeneity in the horizontal distribution of microplastics in the soil affect the productivity (biomass) and species composition of the experimental plant communities? (2) Do the impacts of such soil microplastic heterogeneity on plant communities vary depending on the chemical composition and morphology of the microplastics?

## Materials and methods

### Plant species, microplastics and soil

Experimental plant communities were established by sowing seeds of six perennial grassland species of three functional groups, i.e., two grasses (*Elymus dahuricus* Turcz., *Lolium perenne* L.), two legumes (*Medicago sativa* L. and *Trifolium repens* L.) and two forbs (*Plantago asiatica* L. and *Taraxacum mongolicum* Hand.-Mazz.). Seeds of all plant species were purchased from Jiangsu Leerda Seed Industry Co., LTD., in Xuzhou, Jiangsu Province, China, and stored at 4°C before use to keep their vitality.

We used six types of microplastics, i.e., polystyrene foam (EPS; average diameter: 200 μm), polyethylene fiber (PET; average length: 300 μm; specific gravity: 1.36; diameter: 20 μm ± 4 μm), polyethylene bead (HDPE; average diameter: 150 μm), polyethylene fiber (PP; average length: 300 μm; specific gravity: 0.91; diameter: 18 μm ~ 48 μm), polylactic bead (PLA; average diameter: 150 μm) and polyamide bead (PA6; average diameter: 150 μm). EPS is a foam, PET and PP are fibers, and HDPE, PLA and PA6 are beads. These microplastics vary in size, appearance, physical and chemical properties. They are all common plastic pollutants and have been examined in previous studies (Karamanlioglu et al., 2017; Zhou et al., 2018; Lozano et al., 2021). HDPE, PP, PLA and PA6 were purchased from Guangdong Huachuang Plastic Chemical Co., LTD, and PET and PP were purchased from Hunan Huixiang Fiber Co., LTD.

The soil used was a 1:1 (v:v) mixture of river sand and a local soil collected in Taizhou, Zhejiang, China. The local soil was sieved to pass 2-cm mesh to remove gravels and plant debris. The soil mixture contained organic carbon of 2.11 g kg^-1^, total nitrogen of 0.07 g kg^-1^ and total phosphorus of 0.91 g kg^-1^.

### Experimental design

**Figure 1:**
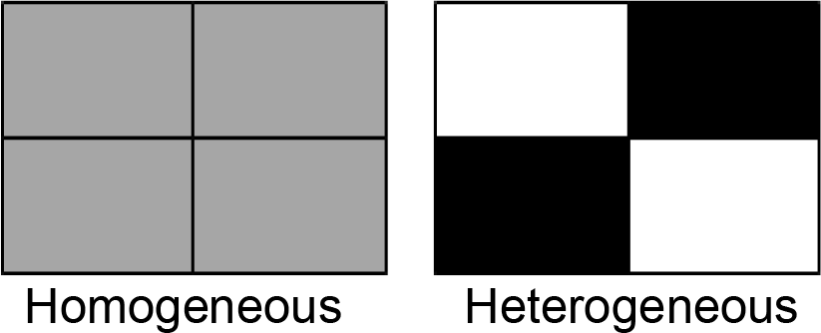
Schematic representation of the experimental design. For each of the six types of microplastics, we established a homogeneous and a heterogeneous soil treatment. For the homogeneous treatment, each of the four equal patches in a box was filled with the soil uniformly mixed with a low concentration (0.1%) of microplastics (grey); for the heterogeneous treatment, two opposite patches in a box were filled with the soil without microplastics (white) and the other two with the soil uniformly mixed with a higher concentration (0.2%) of microplastics (black). The total amounts of microplastics in the homogeneous and heterogeneous treatments were the same.

For each of the six microplastics, we first created three types of soils using the soil mixture described above and the microplastic: (1) a blank soil without any microplastics, (2) a soil containing 0.1% (i.e., 1g kg^-1^) of the microplastic (low-concentration soil) and (3) a soil containing 0.2% of the microplastic (high-concentration soil). The concentrations of microplastics used in this study were within the range of microplastic concentrations in soils collected in the field (Blasing and Amelung, 2018; Zhang and Liu, 2018) and were also used in previous studies (de Souza Machado et al., 2018b). For each microplastic, we established two soil treatments (homogeneous vs. heterogeneous) in boxes (38 cm long × 28 cm wide × 14 cm deep). Each box was divided into four equal patches (19 cm long × 14 cm wide × 14 cm deep) by a plastic divider. For the homogeneous soil treatment (control), each of the four patches in a box was filled with the low-concentration soil; for the heterogeneous soil treatment, two opposite patches in a box were filled with the blank soil and the other two with the high-concentration soil (**Figure 1**). After filling the soils, we removed the divider from the box so that plant roots could grow freely across patches. Each treatment was replicated six times, resulting in a total of 72 boxes (6 microplastics × 2 soil treatments × 6 replicates).

Plant communities were established by directly sowing seeds into each box. For each of the four patches in a box, we sowed about 54 seeds for each of the six species, resulting in a total density of 2000 seeds/m^2^ in the whole box. Within each patch, the seeds of each of the six species were roughly evenly distributed.

All boxes were randomly placed in a greenhouse of Taizhou University in Taizhou, Zhejiang Province, China. The experiment started on 1 September 2020, and ended on 1 March 2021. Sufficient water was supplied to each box every 1-2 days to keep the soil moist.

### Measurements and analyses

At the end of the experiment, we harvested the aboveground parts of each species in each patch in each box. Plant roots in each patch were also harvested, but it was not possible to sort them into species. After clearing, all plant parts were oven-dried at 70 °C for 72 hours and weighed. In the heterogeneous treatment, in each box plants in the two opposite soil patches without microplastics (referred to as high-quality patches) were pooled and those in the two soil patches with microplastics (referred to as low-quality patches) were pooled. In the homogeneous treatment, plants were treated in the similar way as those in the heterogeneous treatment for the purpose of analysis, i.e., in each box two opposite patches were referred to as imagined high-quality patches and the other two as imagined low-quality patches.

We calculated evenness of the plant community in each box based on aboveground biomass of each plant species (Pielou, 1966; Xue et al., 2021). Two-way ANOVA was used to test the effects of microplastic type (EPS, PET, PP, HDPE, PLA and PA6), soil heterogeneity (homogeneous vs. heterogeneous) and their interaction on root, shoot and total biomass and species evenness of the plant communities at the whole box level. Following two-way ANOVA, linear contrasts were used to test whether mean values differed significantly between the homogeneous and the heterogeneous treatment within each type of microplastics (Sokal and Rohlf, 1995). Three-way ANOVA was used to examined the effects of microplastic type, soil heterogeneity and patch type (low- vs. high-quality patches) on root, shoot and total biomass of the plant communities at the patch level. Box identity was included as a random factor as the data from the two types of patches in a box were not independent. Following three-way ANOVA, linear contrasts were used to test whether mean values differed significantly between the high- and the low-quality patches within each of 12 combinations of the microplastic type and soil heterogeneity treatments.

We also analyzed the effects of microplastic shape (foam, fiber and bead) and soil heterogeneity on biomass and evenness of the plant communities at the whole box level, and the effects of microplastic shape, soil heterogeneity and patch quality on biomass of the plant communities at the patch level. In these analysis, microplastic type (EPS, PET, PP, HDPE, PLA and PA6) and/or box identity were included as random factors. Before analysis, the data were tested for normality and homogeneity. The analyses were implemented using IBM SPSS 23.0 (IBM Corp., Armonk, NY, USA) and R (version 4.1.2; http://www.r-project.org) in RStudio (version 2021.09.1 Build 372; https://www.rstudio.com/).

## Results

### Effects on biomass of plant communities at the whole box level

**Figure 2:**
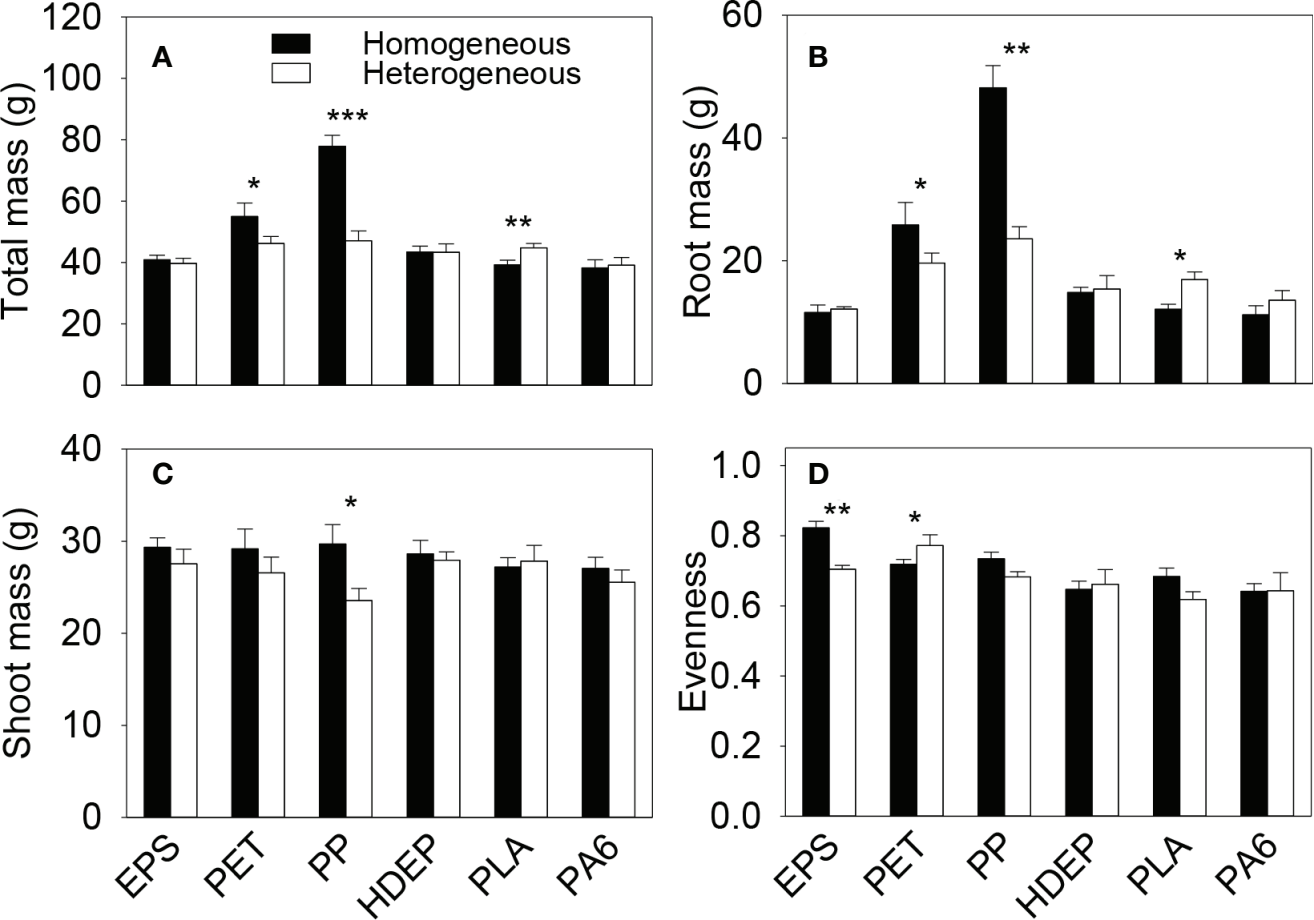
**(A)** Total, **(B)** root and **(C)** shoot biomass and **(D)** species evenness of the plant communities in the homogeneous and the heterogeneous treatment for each of the six types of microplastics. Bars and vertical lines are mean and SE. Symbols (^***^ *P* < 0.001, ^**^ *P* < 0.01 and ^*^ *P* < 0.05) indicate significant differences between the homogeneous and the heterogeneous treatment.

**Figure 3:**
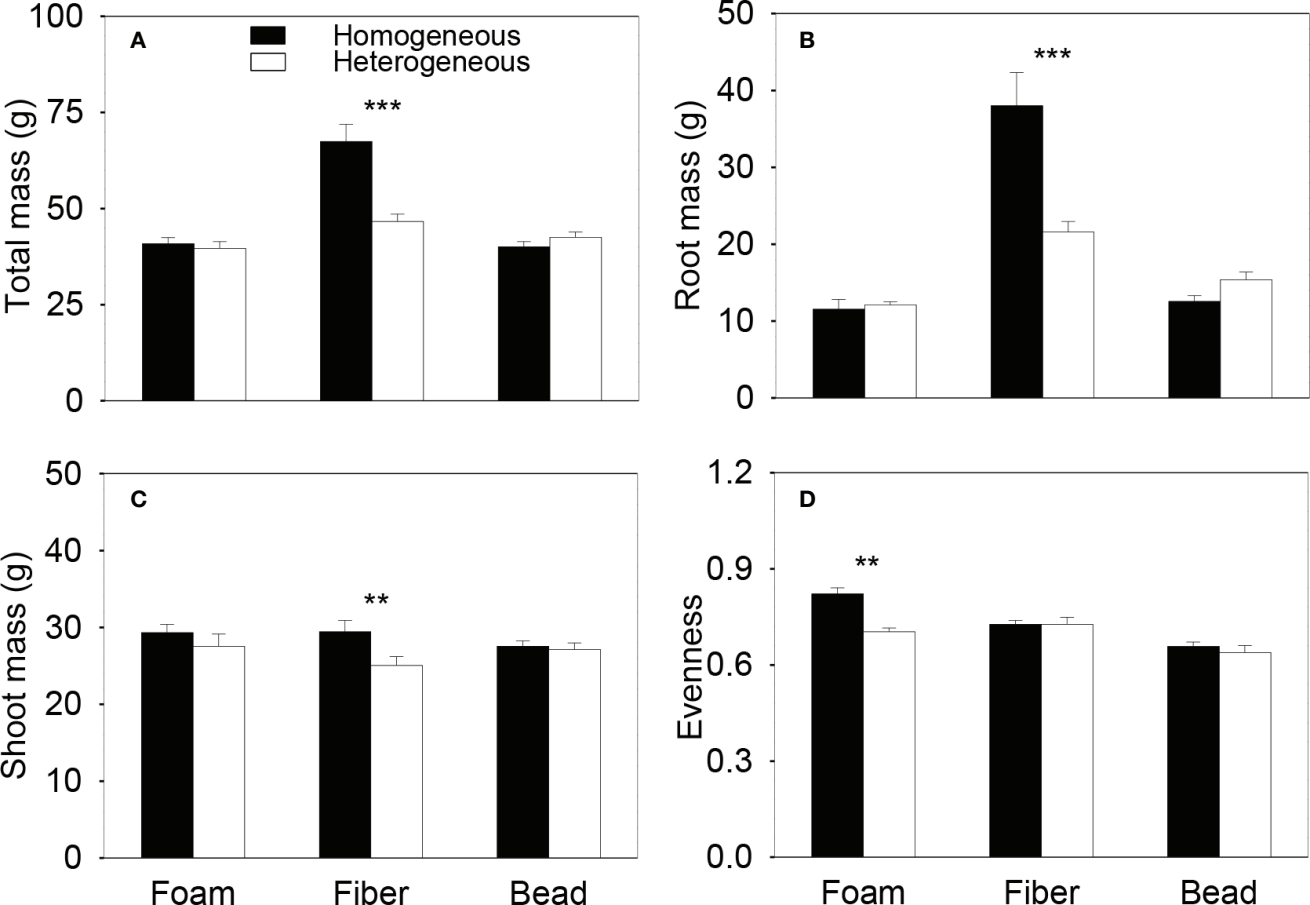
**(A)** Total, **(B)** root and **(C)** shoot biomass and **(D)** species evenness of the plant communities in the homogeneous and the heterogeneous treatment for each of the three shapes of microplastics. Bars and vertical lines are mean and SE. Symbols (^***^ *P* < 0.001 and ^**^ *P* < 0.01) indicate significant differences between the homogeneous and the heterogeneous treatment.

Total biomass and root biomass of the plant communities in the whole boxes were significantly higher in the homogeneous than in the heterogeneous treatment when the microplastic type was PET and PP, were significantly smaller when the microplastic type was PLA, but were not significantly different when the microplastic type was EPS, HDPE or PA6 (**Figure 2A, B**, **Table S1**). Shoot biomass of the whole communities was significantly higher in the homogeneous than in the heterogeneous treatment when the microplastic type was PP, but showed no significant difference between the homogeneous and the heterogeneous treatment for the other five types of microplastics (**Figure 2C**, **Table S1**). Total, root and shoot biomass of the whole plant communities were all significantly higher in the homogeneous than in the heterogeneous treatment when the microplastic shape was fiber, but did not differ between the two soil treatments when the microplastic shape was foam or bead (**Figure 3**, **Table S2**).

### Effects on biomass of plant communities at the patch level

**Figure 4:**
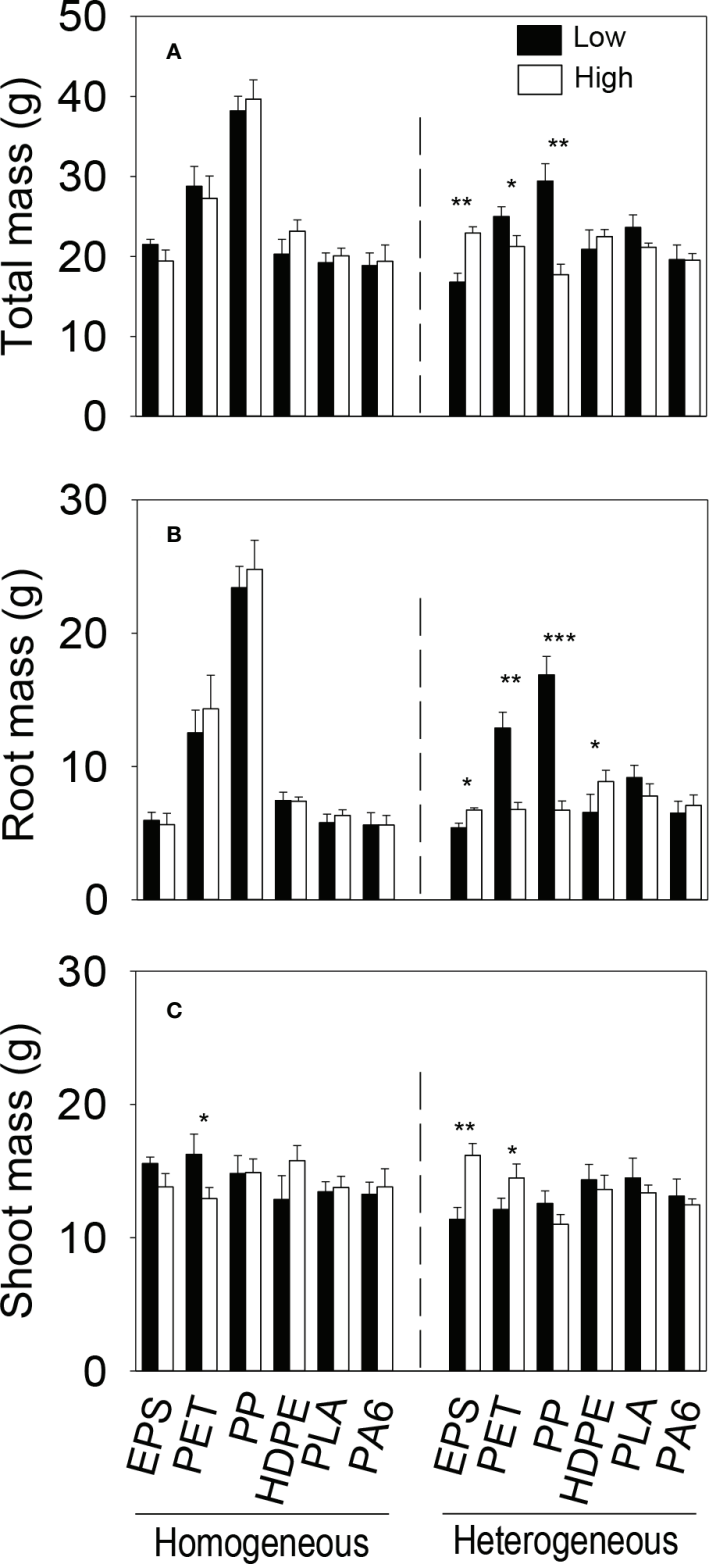
**(A)** Total, **(B)** root and **(C)** shoot biomass of the plant communities in the (imagined) high- and low-quality patches in the homogeneous and the heterogeneous treatment for each of the six microplastics. Bars and vertical lines are mean and SE. Symbols (^***^ *P* < 0.001, ^**^ *P* < 0.01 and ^*^ *P* < 0.05) indicate significant differences between the high- and low-quality patches.

We observed significant three-way interaction effects of both microplastic type × soil heterogeneity × patch quality and microplastic shape × soil heterogeneity × patch quality on all three biomass measures at the patch level (**Table S3-S4**). At the patch level, biomass (total, root and shoot) of the plant communities generally did not differ significantly between the imagined two types of patches in the homogeneous treatment in any of the six microplastic types or in any of the three microplastic shapes (foam, fiber and bead); the only exception was shoot biomass which was higher in the low- than in the high-quality patches in the homogeneous treatment when the microplastic type was PET (**Figure 4C**).

**Figure 5:**
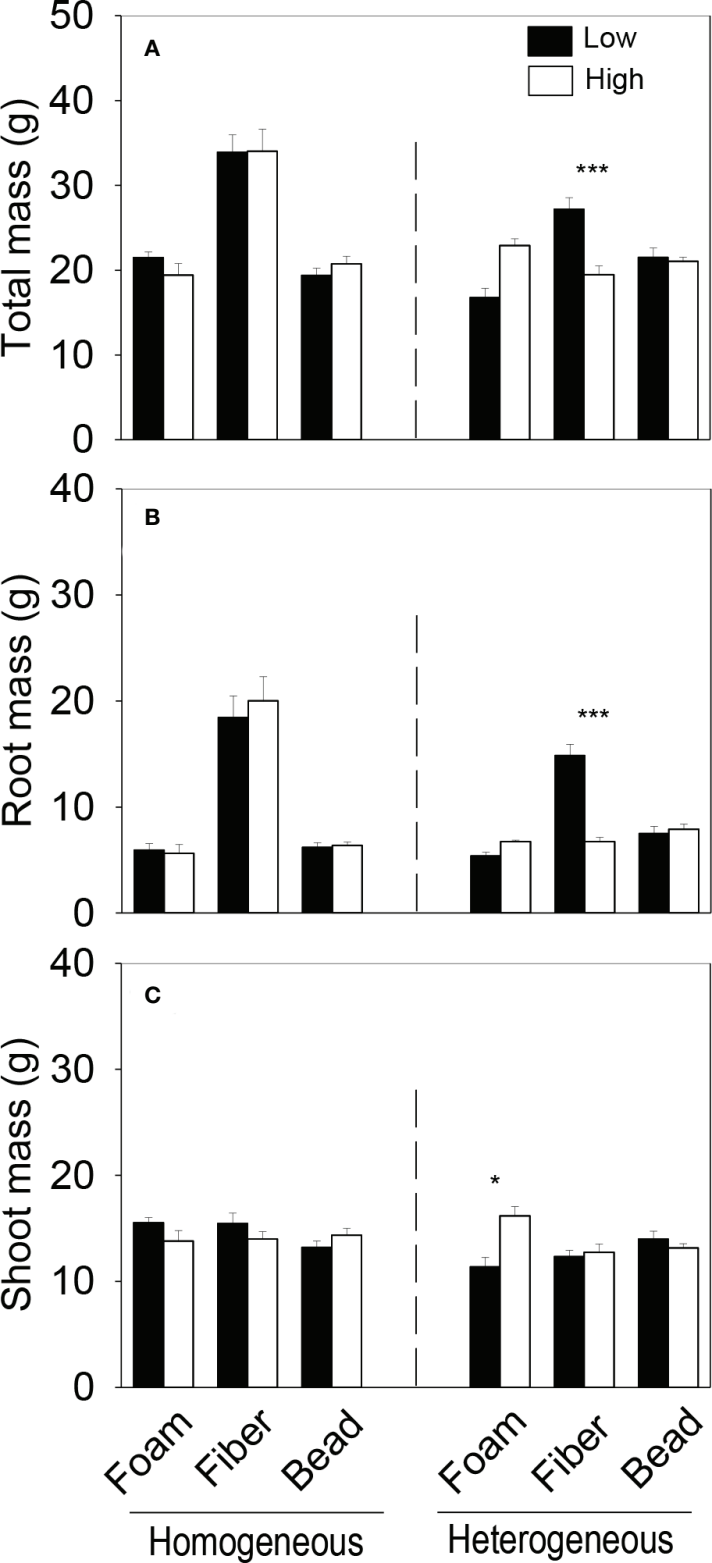
**(A)** Total, **(B)** root and **(C)** shoot biomass of the plant communities in the (imagined) high- and low-quality patches in the homogeneous and the heterogeneous treatment for each of the thee microplastic shapes. Bars and vertical lines are mean and SE. Symbols (^***^ *P* < 0.001 and ^*^ *P* < 0.05) indicate significant differences between the high-and low-quality patches.

In the heterogeneous treatment, however, total biomass and root biomass of the plant communities were significantly smaller in the low-quality patches (with the higher concentration of microplastics) than in the high-quality patches (without microplastics) when the microplastic type was EPS, but greater when the microplastic type was PET or PP (**Figures 4A, B**). In addition, in the heterogeneous treatment, root biomass was significantly smaller in the low- than in the high-quality patches when the microplastic was HDPE (**Figure 4B**), and shoot biomass was also significantly smaller when the microplastic was EPS and PET (**Figure 4C**). In the heterogeneous treatment, total and root biomass of the plant communities were significantly smaller in the high- than in the low-quality soil patches when the microplastic shape was fiber, but did not differ between the two types of patches when the microplastic shape was foam or bead (**Figures 5A, B**). Shoot biomass of the plant communities was significantly higher in the high- than in the low-quality patches when the microplastic shape was foam, but showed no difference between the patch types when the microplastic shape was fiber or bead (**Figure 5C**)

### Effects on species diversity of plant communities at the whole box level

Species evenness of the plant communities was significantly higher in the homogeneous than in the heterogeneous treatment when the microplastic type was EPS, was significantly lower when the microplastic type was PET, but showed no significant difference when the microplastic type was HDPE, PP, PLA or PA6 (**Figure 2D**, **Table S1**). Species evenness was also significantly higher in the homogeneous than in the heterogeneous treatment when the microplastic shape was foam, but showed no difference between the two soil treatments when it was fiber or bead (**Figure 3D**, **Table S2**)

## Discussion

Contamination of soils by microplastics can have profound ecological impacts on terrestrial ecosystems (Rillig, 2012; de Souza Machado et al., 2018a; Roy et al., 2022). While previous studies have shown that microplastics in soils may influence plant growth and community productivity and composition (de Souza Machado et al., 2019; Rillig et al., 2019; Lozano and Rillig, 2020), no study has tested the impact of spatial heterogeneity of soil microplastics. Our study showed for the first time that spatial heterogeneity of soil microplastics could influence productivity and species composition of experimental plant communities, but such effects varied depending on microplastic types and shapes.

When growing in spatially heterogeneous environments consisting of favorable and unfavorable patches, many plants are able to allocate more roots and/or shoots in favorable patches and less in unfavorable patches (Hutchings et al., 2003; Dong et al., 2015; Liu et al., 2020), and such a foraging response may help them take up more resources and increase their growth (Rajaniemi and Reynolds, 2004; Questad and Foster, 2008; Xue et al., 2020). We also found that the plant communities showed patch-level foraging responses in the environments with the spatially heterogeneous distribution of soil microplastics. However, such an effect varied with microplastic types and shapes likely due to their different effects on soil physico-chemical properties and soil microbial abundance and activities (de Souza Machado et al., 2018b; Qi et al., 2018; Rillig et al., 2019). Additionally, the effect of soil microplastic heterogeneity on community productivity was not related to the patch-level foraging responses of the plant communities.

EPS and HDPE commonly have a negative effect on plant growth because they can induce cytogenotoxicity by aggravating reactive oxygen species generation (Jiang et al., 2019; Maity and Pramanick, 2020; Pignattelli et al., 2020). We observed that, in the environment with the spatially heterogeneous distribution of soil microplastics, plant communities produced more root, shoot and total biomass in the soil patches without microplastics (high-quality patches) than in the soil patches with the higher (0.2%) concentration of microplastics (low-quality patches) when the microplastic was EPS and more root biomass when the microplastic was HDPE (**Figure 4**), demonstrating root and/or shoot foraging responses (2015; James et al., 2009; Giehl and von Wirén, 2014; Keser et al., 2014). However, such foraging responses at the patch level did not cascade to influence biomass of the plant communities at the whole box level (**Figures 2A-C**), as also reported in some previous studies testing the effect of spatial heterogeneity in the distribution of other soil factors (Dong et al., 2015; Liu et al., 2017; Xue et al., 2018; Adomako et al., 2021b; Yao et al., 2021).

PET and PP used in this study are both microplastic fibers. Adding plastic fibers to the soil can reduce soil bulk density (Rillig et al., 2019), increase soil porosity (Zhao et al., 2021) and permeability (de Souza Machado et al., 2019), which can facilitate plants to take roots into the soil (Zimmerman and Kardos, 1961). Thus, in the heterogeneous treatment with PET and PP, root biomass was greatly improved when plants grew in the patches with a higher concentration (0.2%) of PET or PP than in the patches without microplastics (**Figure 4B**), resulting in higher total biomass of the plant communities in the PET and PP patches (**Figure 4A**). Consequently, total and root biomass of the plant communities also differed greatly between the two types of soil patches when the microplastic shape was fiber (**Figures 5A, B**). These results suggest that the plant communities also demonstrated foraging responses in the soil with the spatially heterogeneous distribution of microplastic fibers such as PET and PP.

However, such foraging responses did not result in promoted growth of the whole plant communities (at the whole box level). Instead, the spatially heterogeneous distribution of microplastic fibers (PET and PP) in the soil decreased biomass of the whole plant community biomass (**Figures 2A-C**, **3A-C**). The decreased growth was because plants grew better when the soil contained 0.1% of PET and PP in the homogeneous treatment than when the soil did not contain any microplastics or contained the higher concentration (0.2%) of microplastics in the heterogeneous treatment (all *P* < 0.5; **Figures 3A, B**). This result suggests that the positive effect of microplastic fibers on plant growth can vary depending on their concentrations in the soil (Lozano et al., 2021).

At the whole box level, we observed that the plant communities produced more total and root biomass in the soil with the heterogeneous distribution of PLA than in the soil with the homogeneous distribution (**Figures 2A, B**). However, the plant communities did not show patch-level foraging responses in the soil with the heterogeneous distribution of PLA (**Figure 4**) or in the soil with the heterogeneous distribution of microplastic beads in general (**Figure 5**), suggesting that this benefit of soil microplastic heterogeneity was not related to foraging responses (Adomako et al., 2020; Liu et al., 2020; Cao et al., 2022). PLA is one type of biodegradable microplastics, and can change soil physico-chemical properties such as soil pH in the initial stage of polylactic acid degradation, which may affect soil microbial communities (Karamanlioglu et al., 2017; Chamas et al., 2020). Previous studies showed that PLA can negatively affect plant growth (Souza et al., 2013; Boots et al., 2019). In this study, the promoted community growth in the soil with the heterogeneous distribution of PLA was solely because the plant communities grew worse when the soil contained 0.1% of PLA (in the homogeneous treatment) than when it did not contain PLA or contained the higher concentration (0.2%; in the heterogeneous treatment), particularly for root growth (all *P* < 0.05; **Figure 4B**). This result suggests that the negative impact of PLA on plant growth can vary depending on its concentration in the soil, as reported for other microplastics (van Kleunen et al., 2020; Lozano et al., 2021).

A plant community commonly comprises species of different foraging abilities and consequently environmental heterogeneity may alter its species composition because it may benefit species with a higher foraging ability more than those with a low foraging ability or those do not demonstrate the foraging response (Hutchings et al., 2003; Xue et al., 2020). In our study, spatial heterogeneity of soil microplastics decreased species evenness when the microplastic was EPS (with a foam shape), promoted it when the microplastic was PET (with a fiber shape), and had no effect when the microplastic was one of the other four microplastics (with either a fiber or a bead shape; **Figure 2D**). These results suggest that spatial heterogeneity in the horizontal distribution of microplastics in the soil can affect species composition of plant communities, but such an effect varies depending on microplastic types and likely also microplastic shapes. It is well-known that different types and shapes of microplastics may differentially affect plant growth because they may differ in phytotoxicity (Dong et al., 2020; Pignattelli et al., 2020; Li et al., 2022) and in the effect on soil physio-chemical properties and soil microbial communities such as soil microbial activity and mycorrhizal binding in plant roots (de Souza Machado et al., 2019; Rillig et al., 2019; Lozano and Rillig, 2020; Lozano et al., 2021; Zhao et al., 2021). The promoted evenness of soil heterogeneity in the horizontal distribution of microplastics may be due to the increased microhabitat diversity, as observed in studies examining effects of soil heterogeneity in other factors (Liu et al., 2021; Helbach et al., 2022). However, it is unclear what resulted in the decreased species evenness in the soil with the heterogeneous distribution of EPS, which seemed not to be related to the difference in the patch-level responses of individual plant species (**Figure S1**, **Table S5**). Further studies could be designed to resolve this question.

In our experiment, there were no physical barriers between patches with and without microplastics. This setup mimicked the situation in the field that allowed plant roots to grow freely across adjacent patches. In a long run, plant growth, soil biota activity and water movement will eventually homogenize the soil in the container that is heterogeneous at the beginning. However, this process usually will take a much long time compared to the shorter experimental duration in the greenhouse, which will not influence the treatment effect.

We conclude that soil heterogeneity in microplastics can influence productivity and species composition of plant communities, but such an effect varies depending on microplastic types and likely also shapes (e.g., chemical composition and morphology). However, our results fail to support the idea that foraging responses of plant communities can result in promoted productivity. Further studies could combine soil microbial and physico-chemical analyses to explore the mechanisms underlying the effect of soil microplastic heterogeneity on community productivity (Baer et al., 2020; Lozano et al., 2021; Xue et al., 2021; Zhao et al., 2021). The impacts of patch scale and patch contrast of soil microplastic heterogeneity should also be considered in future (Adomako et al., 2021b; Li et al., 2022).

## Data Availability

The raw data supporting the conclusions of this article will be made available by the authors, without undue reservation.

## References

[B1] Adomako M. O. Gao F. L. Li J. M. Du D. L. Xue W. Yu F. H. (2020). Effects of soil nutrient heterogeneity and parasitic plant infection on an experimental grassland community. Flora 271, 151666. doi: 10.1016/j.flora.2020.151666

[B2] Adomako M. O. Xue W. Du D. L. Yu F. H. (2021a). Soil biota and soil substrates influence responses of the rhizomatous clonal grass *Leymus chinensis* to nutrient heterogeneity. Plant Soil 465, 19–29. doi: 10.1007/s11104-021-04967-0

[B3] Adomako M. O. Xue W. Roiloa S. Zhang Q. Du D. L. Yu F. H. (2021b). Earthworms modulate impacts of soil heterogeneity on plant growth at different spatial scales. Front. Plant Sci. 12, 735495. doi: 10.3389/fpls.2021.735495 35003149 PMC8732864

[B4] Baer S. G. Adams T. Scott D. A. Blair J. M. Collins S. L. (2020). Soil heterogeneity increases plant diversity after 20 years of manipulation during grassland restoration. Ecol. Appl. 30, e02014. doi: 10.1002/eap.2014 31587410

[B5] Baer S. G. Collins S. L. Blair J. M. Knapp A. K. Fiedler A. K. (2005). Soil heterogeneity effects on tallgrass prairie community heterogeneity: An application of ecological theory to restoration ecology. Restor. Ecol. 13, 413–424. doi: 10.1111/j.1526-100X.2005.00051.x

[B6] Barnes D. K. Galgani F. Thompson R. C. Barlaz M. (2009). Accumulation and fragmentation of plastic debris in global environments. Philos. Trans. R. Soc. B: Biol. Sci. 364, 1985–1998. doi: 10.1098/rstb.2008.0205 PMC2873009 19528051

[B7] Blasing M. Amelung W. (2018). Plastics in soil: Analytical methods and possible sources. Sci. Total Environ. 612, 422–435. doi: 10.1016/j.scitotenv.2017.08.086 28863373

[B8] Boots B. Russell C. W. Green D. S. (2019). Effects of microplastics in soil ecosystems: Above and below ground. Environ. Sci. Technol. 53, 11496–11506. doi: 10.1021/acs.est.9b03304 31509704

[B9] Bosker T. Bouwman L. J. Brun N. R. Behrens P. Vijver M. G. (2019). Microplastics accumulate on pores in seed capsule and delay germination and root growth of the terrestrial vascular plant *Lepidium sativum* . Chemosphere 226, 774–781. doi: 10.1016/j.chemosphere.2019.03.163 30965248

[B10] Cai L. Q. Wang J. D. Peng J. P. Tan Z. Zhan Z. W. Tan X. L.. (2017). Characteristic of microplastics in the atmospheric fallout from dongguan city, China: preliminary research and first evidence. Environ. Sci. pollut. Res. 24, 24928–24935. doi: 10.1007/s11356-017-0116-x 28918553

[B11] Cao X. X. Xue W. Lei N. F. Yu F. H. (2022). Effects of clonal integration on foraging behavior of three clonal plants in heterogeneous soil environments. Forests 13, 696. doi: 10.3390/f13050696

[B12] Chamas A. Moon H. Zheng J. J. Qiu Y. Tabassum T. Jang J. H.. (2020). Degradation rates of plastics in the environment. ACS Sustain. Chem. Eng. 8, 3494–3511. doi: 10.1021/acssuschemeng.9b06635

[B13] Chen D. Ali A. Yong X. H. Lin C. G. Niu X. H. Cai A. M.. (2019). A multi-species comparison of selective placement patterns of ramets in invasive alien and native clonal plants to light, soil nutrient and water heterogeneity. Sci. Total Environ. 657, 1568–1577. doi: 10.1016/j.scitotenv.2018.12.099 30677922

[B14] Corradini F. Meza P. Eguiluz R. Casado F. Huerta-Lwanga E. Geissen V. (2019). Evidence of microplastic accumulation in agricultural soils from sewage sludge disposal. Sci. Total Environ. 671, 411–420. doi: 10.1016/j.scitotenv.2019.03.368 30933797

[B15] Cunha C. Lopes J. Paulo J. Faria M. Kaufmann M. Nogueira N.. (2020). The effect of microplastics pollution in microalgal biomass production: A biochemical study. Water Res. 186, 116370. doi: 10.1016/j.watres.2020.116370 32906034

[B16] de Souza Machado A. A. Kloas W. Zarfl C. Hempel S. Rillig M. C. (2018a). Microplastics as an emerging threat to terrestrial ecosystems. Global Change Biol. 24, 1405–1416. doi: 10.1111/gcb.14020 PMC5834940 29245177

[B17] de Souza Machado A. A. Lau C. W. Kloas W. Bergmann J. Bachelier J. B. Faltin E.. (2019). Microplastics can change soil properties and affect plant performance. Environ. Sci. Technol. 53, 6044–6052. doi: 10.1021/acs.est.9b01339 31021077

[B18] de Souza Machado A. A. Lau C. W. Till J. Kloas W. Lehmann A. Becker R.. (2018b). Impacts of microplastics on the soil biophysical environment. Environ. Sci. Technol. 52, 9656–9665. doi: 10.1021/acs.est.8b02212 30053368 PMC6128618

[B20] Dong Y. M. Gao M. L. Song Z. G. Qiu W. W. (2020). Microplastic particles increase arsenic toxicity to rice seedlings. Environ. pollut. 259, 113892. doi: 10.1016/j.envpol.2019.113892 31931412

[B19] Dong B. C. Wang J. Z. Liu R. H. Zhang M. X. Luo F. L. Yu F. H. (2015). Soil heterogeneity affects ramet placement of *Hydrocotyle vulgaris* . J. Plant Ecol. 8, 91–100. doi: 10.1093/jpe/rtu003

[B21] Gao F. L. He Q. S. Zhang Y. D. Hou J. H. Yu F. H. (2021). Effects of soil nutrient heterogeneity on the growth and invasion success of alien plants: A multi-species study. Front. Ecol. Evol. 8, 619861. doi: 10.3389/fevo.2020.619861

[B22] Giehl R. F. von Wirén N. (2014). Root nutrient foraging. Plant Physiol. 166, 509–517. doi: 10.1104/pp.114.245225 25082891 PMC4213083

[B23] Helbach J. Frey J. Messier C. Mörsdorf M. Scherer-Lorenzen M. (2022). Light heterogeneity affects understory plant species richness in temperate forests supporting the heterogeneity-diversity hypothesis. Ecol. Evol. 12, e8534. doi: 10.1002/ece3.8534 35222947 PMC8858222

[B26] Huang L. Dong B. C. Xue W. Peng Y. K. Zhang M. X. Yu F. H. (2013). Soil particle heterogeneity affects the growth of a rhizomatous wetland plant. PloS One 8, e69836. doi: 10.1371/journal.pone.0069836 23936110 PMC3728365

[B25] Huang D. Wang X. Yin L. Chen S. Tao J. Zhou W.. (2021). Research progress of microplastics in soil-plant system: Ecological effects and potential risks. Sci. Total Environ. 812, 151487. doi: 10.1016/j.scitotenv.2021.151487 34742990

[B27] Hutchings M. J. John E. A. Wijesinghe D. K. (2003). Toward understanding the consequences of soil heterogeneity for plant populations and communities. Ecology 84, 2322–2334. doi: 10.1890/02-0290

[B24] Hu Y. Xu Z. W. Li M. Y. Croy J. R. Zhang Z. Y. Li H. M.. (2022). Increasing soil heterogeneity strengthens the inhibition of a native woody plant by an invasive congener. Plant Soil. doi: 10.1007/s11104-022-05666-0

[B28] James J. J. Mangold J. M. Sheley R. L. Svejcar T. (2009). Root plasticity of native and invasive great basin species in response to soil nitrogen heterogeneity. Plant Ecol. 202, 211–220. doi: 10.1007/s11258-008-9457-3

[B29] Jiang X. F. Chen H. Liao Y. C. Ye Z. Q. Li M. Klobucar G. (2019). Ecotoxicity and genotoxicity of polystyrene microplastics on higher plant *Vicia faba* . Environ. pollut. 250, 831–838. doi: 10.1016/j.envpol.2019.04.055 31051394

[B30] Karamanlioglu M. Preziosi R. Robson G. D. (2017). Abiotic and biotic environmental degradation of the bioplastic polymer polylactic acid: A review. Polym. Degrad. Stab. 137, 122–130. doi: 10.1016/j.polymdegradstab.2017.01.009

[B31] Keser L. H. Dawson W. Song Y. B. Yu F. H. Fischer M. Dong M.. (2014). Invasive clonal plant species have a greater root-foraging plasticity than non-invasive ones. Oecologia 174, 1055–1064. doi: 10.1007/s00442-013-2829-y 24352844

[B32] Keser L. H. Visser E. J. Dawson W. Song Y. B. Yu F. H. Fischer M.. (2015). Herbaceous plant species invading natural areas tend to have stronger adaptive root foraging than other naturalized species. Front. Plant Sci. 6, 273. doi: 10.3389/fpls.2015.00273 25964790 PMC4410514

[B34] Liang J. F. Yuan W. Y. Gao J. Q. Roiloa S. R. Song M. H. Zhang X. Y.. (2020). Soil resource heterogeneity competitively favors an invasive clonal plant over a native one. Oecologia 193, 155–165. doi: 10.1007/s00442-020-04660-6 32356013

[B35] Liu L. Alpert P. Dong B. C. Li J. M. Yu F. H. (2017). Combined effects of soil heterogeneity, herbivory and detritivory on growth of the clonal plant *Hydrocotyle vulgaris* . Plant Soil 421, 429–437. doi: 10.1007/s11104-017-3476-6

[B36] Liu L. Alpert P. Dong B. C. Yu F. H. (2020). Modification by earthworms of effects of soil heterogeneity and root foraging in eight species of grass. Sci. Total Environ. 708, 134941. doi: 10.1016/j.scitotenv.2019.134941 31796271

[B37] Liu Y. Qi W. C. He D. N. Xiang Y. R. Liu J. C. Huang H. M.. (2021). Soil resource availability is much more important than soil resource heterogeneity in determining the species diversity and abundance of karst plant communities. Ecol. Evol. 11, 16680–16692. doi: 10.1002/ece3.8285 34938465 PMC8668789

[B33] Li J. Yu S. G. Yu Y. F. Xu M. L. (2022). Effects of microplastics on higher plants: A review. Bull. Environ. Contam. Toxicol. 109, 241–265. doi: 10.1007/s00128-022-03566-8 35752996

[B38] Lozano Y. M. Lehnert T. Linck L. T. Lehmann A. Rillig M. C. (2021). Microplastic shape, polymer type, and concentration affect soil properties and plant biomass. Front. Plant Sci. 12, 616645. doi: 10.3389/fpls.2021.616645 33664758 PMC7920964

[B39] Lozano Y. M. Rillig M. C. (2020). Effects of microplastic fibers and drought on plant communities. Environ. Sci. Technol. 54, 6166–6173. doi: 10.1021/acs.est.0c01051 32289223 PMC7241422

[B40] Lwanga E. H. Gertsen H. Gooren H. Peters P. Salanki T. van der Ploeg M.. (2017). Incorporation of microplastics from litter into burrows of *Lumbricus terrestris* . Environ. pollut. 220, 523–531. doi: 10.1016/j.envpol.2016.09.096 27726978

[B41] Maity S. Pramanick K. (2020). Perspectives and challenges of micro/nanoplastics-induced toxicity with special reference to phytotoxicity. Global Change Biol. 26 (6), 3241–3250. doi: 10.1111/gcb.15074 32153083

[B42] Nizzetto L. Bussi G. Futter M. N. Butterfield D. Whitehead P. G. (2016). A theoretical assessment of microplastic transport in river catchments and their retention by soils and river sediments. Environ. Sci.-Process. Impacts 18, 1050–1059. doi: 10.1039/C6EM00206D 27255969

[B43] Pielou E. C. (1966). The measurement of diversity in different types of biological collections. J. Theor. Biol. 13, 131–144. doi: 10.1016/0022-5193(66)90013-0

[B44] Pignattelli S. Broccoli A. Renzi M. (2020). Physiological responses of garden cress (*L. sativum*) to different types of microplastics. Sci. Total Environ. 727, 138609. doi: 10.1016/j.scitotenv.2020.138609 32339829

[B45] Qi Y. L. Yang X. M. Pelaez A. M. Lwanga E. H. Beriot N. Gertsen H.. (2018). Macro-and micro-plastics in soil-plant system: Effects of plastic mulch film residues on wheat (*Triticum aestivum*) growth. Sci. Total Environ. 645, 1048–1056. doi: 10.1016/j.scitotenv.2018.07.229 30248830

[B46] Questad E. J. Foster B. L. (2008). Coexistence through spatio-temporal heterogeneity and species sorting in grassland plant communities. Ecol. Lett. 11, 717–726. doi: 10.1111/j.1461-0248.2008.01186.x 18445035

[B47] Rajaniemi T. K. Reynolds H. L. (2004). Root foraging for patchy resources in eight herbaceous plant species. Oecologia 141, 519–525. doi: 10.1007/s00442-004-1666-4 15278432

[B48] Rillig M. C. (2012). Microplastic in terrestrial ecosystems and the soil? Environ. Sci. Technol. 46, 6453–6454. doi: 10.1021/es302011r 22676039

[B49] Rillig M. C. Ingraffia R. Machado A. A. D. (2017). Microplastic incorporation into soil in agroecosystems. Front. Plant Sci. 8, 1805. doi: 10.3389/fpls.2017.01805 29093730 PMC5651362

[B50] Rillig M. C. Lehmann A. de Souza Machado A. A. Yang G. (2019). Microplastic effects on plants. New Phytol. 223, 1066–1070. doi: 10.1111/nph.15794 30883812

[B51] Roiloa S. R. Retuerto R. (2012). Clonal integration in *Fragaria vesca* growing in metal-polluted soils: Parents face penalties for establishing their offspring in unsuitable environments. Ecol. Res. 27, 95–106. doi: 10.1007/s11284-011-0876-6

[B52] Roy P. Mohanty A. K. Misra M. (2022). Microplastics in ecosystems: Their implications and mitigation pathways. Environ. Sci.: Adv. 1, 9–29. doi: 10.1039/D1VA00012H

[B53] Si C. Xue W. Guo Z. W. Zhang J. F. Hong M. M. Wang Y. Y.. (2021). Soil heterogeneity and earthworms independently promote growth of two bamboo species. Ecol. Indic. 130, 108068. doi: 10.1016/j.ecolind.2021.108068

[B54] Sokal R. R. Rohlf F. J. (1995). Biometry: the principles and practice of statistics in biological research (New York: W. H. Freeman and Company).

[B55] Souza P. M. S. Corroque N. A. Morales A. R. Marin-Morales M. A. Mei L. H. I. (2013). PLA and organoclays nanocomposites: degradation process and evaluation of ecotoxicity using *Allium cepa* as test organism. J. Polym. Environ. 21 (4), 1052–1063. doi: 10.1007/s10924-013-0604-0

[B56] Steinmetz Z. Wollmann C. Schaefer M. Buchmann C. David J. Troeger J.. (2016). Plastic mulching in agriculture. trading short-term agronomic benefits for long-term soil degradation? Sci. Total Environ. 550, 690–705. doi: 10.1016/j.scitotenv.2016.01.153 26849333

[B57] Sun Q. H. Li J. Wang C. Chen A. Q. You Y. L. Yang S. P.. (2022). Research progress on distribution,sources,identification,toxicity,and biodegradation of microplastics in the ocean,freshwater,and soil environment. Front. Environ. Sci. Eng. 16, 14. doi: 10.1007/s11783-021-1429-z

[B58] Tsunoda T. Kachi N. Suzuki J.-I. (2014). Interactive effects of soil nutrient heterogeneity and belowground herbivory on the growth of plants with different root foraging traits. Plant Soil 384, 327–334. doi: 10.1007/s11104-014-2215-5

[B59] van Kleunen M. Brumer A. Gutbrod L. Zhang Z. J. (2020). A microplastic used as infill material in artificial sport turfs reduces plant growth. Plants People Planet 2, 157–166. doi: 10.1002/ppp3.10071

[B61] Wang Y. J. Muller-Scharer H. van Kleunen M. Cai A. M. Zhang P. Yan R.. (2017). Invasive alien plants benefit more from clonal integration in heterogeneous environments than natives. New Phytol. 216, 1072–1078. doi: 10.1111/nph.14820 28944478

[B60] Wan Y. Wu C. X. Xue Q. Hui X. M. N. (2019). Effects of plastic contamination on water evaporation and desiccation cracking in soil. Sci. Total Environ. 654, 576–582. doi: 10.1016/j.scitotenv.2018.11.123 30447596

[B62] Wijesinghe D. K. John E. A. Hutchings M. J. (2005). Does pattern of soil resource heterogeneity determine plant community structure? an experimental investigation. J. Ecol. 93, 99–112. doi: 10.1111/j.0022-0477.2004.00934.x

[B64] Xue W. Huang l. Yu F. H. (2016). Spatial heterogeneity in soil particle size: Does it affect the yield of plant communities with different species richness? J. Plant Ecol. 9, 608–615. doi: 10.1093/jpe/rtv082

[B65] Xue W. Huang L. Yu F. H. (2020). Importance of starting points in heterogeneous environments: Interactions between two clonal plants with contrasting spatial architectures. J. Plant Ecol. 13, 323–330. doi: 10.1093/jpe/rtaa018

[B66] Xue W. Huang L. Yu F. H. (2021). Increasing soil configurational heterogeneity promotes plant community evenness through equalizing differences in competitive ability. Sci. Total Environ. 750, 142308. doi: 10.1016/j.scitotenv.2020.142308 33182201

[B67] Xue W. Huang L. Yu F. H. Bezemer T. M. (2018). Intraspecific aggregation and soil heterogeneity: Competitive interactions of two clonal plants with contrasting spatial architecture. Plant Soil 425, 231–240. doi: 10.1007/s11104-018-3578-9

[B63] Xu L. Zhou Z. F. (2017). Physiological integration affects expansion of an amphibious clonal plant from terrestrial to Cu-polluted aquatic environments. Sci. Rep. 7, 43931. doi: 10.1038/srep43931 28272515 PMC5341073

[B68] Yao S. M. Yu J. Zhang L. M. Lei N. F. Xue W. Chen J. S.. (2021). Effects of soil nutrient heterogeneity and earthworms on aboveground biomass of experimental plant communities. Phyton 90, 1259. doi: 10.32604/phyton.2021.014968

[B69] You W. H. Han C. M. Liu C. H. Yu D. (2016). Effects of clonal integration on the invasive clonal plant *Alternanthera philoxeroides* under heterogeneous and homogeneous water availability. Sci. Rep. 6, 1–8. doi: 10.1038/srep29767 27416868 PMC4945919

[B70] Zhang G. S. Liu Y. F. (2018). The distribution of microplastics in soil aggregate fractions in southwestern China. Sci. Total Environ. 642, 12–20. doi: 10.1016/j.scitotenv.2018.06.004 29894871

[B71] Zhang J. J. Wang X. X. Xue W. T. Xu L. Ding W. C. Zhao M.. (2022). Microplastics pollution in soil increases dramatically with long-term application of organic composts in a wheat–maize rotation. J. Cleaner Prod. 356, 131889. doi: 10.1016/j.jclepro.2022.131889

[B72] Zhao T. T. Lozano Y. M. Rillig M. C. (2021). Microplastics increase soil pH and decrease microbial activities as a function of microplastic shape, polymer type, and exposure time. Front. Environ. Sci. 9, 675805. doi: 10.3389/fenvs.2021.675803

[B73] Zhou J. Dong B. C. Alpert P. Li H. L. Zhang M. X. Lei G. C.. (2012). Effects of soil nutrient heterogeneity on intraspecific competition in the invasive, clonal plant *Alternanthera philoxeroides* . Ann. Bot. 109, 813–818. doi: 10.1093/aob/mcr314 22207612 PMC3286281

[B74] Zhou Q. Zhang H. B. Fu C. C. Zhou Y. Dai Z. F. Li Y.. (2018). The distribution and morphology of microplastics in coastal soils adjacent to the bohai Sea and the yellow Sea. Geoderma 322, 201–208. doi: 10.1016/j.geoderma.2018.02.015

[B75] Zimmerman R. Kardos L. (1961). Effect of bulk density on root growth. Soil Sci. 91, 280–288. doi: 10.1097/00010694-196104000-00012

