# Supplementary material for: Soil heterogeneity in the horizontal distribution of microplastics influences productivity and species composition of plant communities
Source: Front Plant Sci. 2022 Dec 8;13:1075007. doi: 10.3389/fpls.2022.1075007 (PMC9772521; doi:10.3389/fpls.2022.1075007)
Supplement: Supplementary file 1 [file DataSheet_1.docx]

**Supplementary materials**

**Table S1** ANOVA results for effects of microplastic type (EPS, PET, PP, HDPE, PLA and PA6), soil heterogeneity and their interaction on biomass and species diversity of plant communities at the whole box level

|  | Microplastic type (M) | |  | Soil heterogeneity (H) | |  | M × H | |
| --- | --- | --- | --- | --- | --- | --- | --- | --- |
| Variable | *F*_5, 55_ | *P* |  | *F*_1, 55_ | *P* |  | *F*_5, 55_ | *P* |
| Total biomass | **25.26** | **<0.001** |  | **14.86** | **<0.001** |  | **13.65** | **<0.001** |
| Root Biomass | **45.86** | **<0.001** |  | **10.68** | **0.002** |  | **15.96** | **<0.001** |
| Shoot biomass | 0.66 | 0.656 |  | **5.24** | **0.026** |  | 1.22 | 0.312 |
| Evenness | **8.02** | **<0.001** |  | 3.35 | 0.073 |  | **2.83** | **0.024** |

**Table S2** ANOVA results for effects of microplastic shape (foam, fiber and bead), soil heterogeneity and their interaction on biomass and species diversity of plant communities at the whole box level

|  | Microplastic shape (S) | |  | Soil heterogeneity (H) | |  | S × H | |
| --- | --- | --- | --- | --- | --- | --- | --- | --- |
| Variable | *F*_2, 3_ | *P* |  | *F*_1, 58_ | *P* |  | *F*_2, 58_ | *P* |
| Total biomass | 6.15 | 0.087 |  | **12.87** | **<0.001** |  | **18.91** | **<0.001** |
| Root Biomass | 5.90 | 0.091 |  | **9.14** | **0.004** |  | **21.04** | **<0.001** |
| Shoot biomass | 0.59 | 0.608 |  | **5.38** | **0.024** |  | 2.16 | 0.125 |
| Evenness | **19.22** | **0.020** |  | 3.35 | 0.073 |  | **3.45** | **0.039** |

**Table S3** ANOVA results for effects of microplastic type (EPS, PET, PP, HDPE, PLA and PA6), soil heterogeneity, patch type and their interactions on biomass of plant communities at the patch level

|  |  | Total biomass | |  | Root biomass | |  | Shoot biomass | |  |
| --- | --- | --- | --- | --- | --- | --- | --- | --- | --- | --- |
| Effect | DF | *F* | *P* |  | *F* | *P* |  | *F* | *P* |  |
| Microplastic type (M) | 5, 55 | **24.13** | **<0.001** |  | **43.22** | **<0.001** |  | 0.66 | 0.656 |  |
| Soil heterogeneity (H) | 1, 55 | **14.88** | **<0.001** |  | **10.90** | **<0.001** |  | **5.24** | **0.026** |  |
| Patch type (P) | 1, 55 | 1.52 | 0.233 |  | **10.36** | **0.002** |  | 0.14 | 0.714 | |
| M × H | 5, 55 | **13.00** | **<0.001** |  | **15.03** | **<0.001** |  | 1.22 | 0.312 |  |
| M × P | 5, 55 | **4.49** | **0.002** |  | **10.93** | **<0.001** |  | 0.79 | 0.563 |  |
| H × P | 1, 55 | 3.57 | 0.064 |  | **29.11** | **<0.001** |  | 0.72 | 0.401 |  |
| M × H × P | 5, 55 | **6.64** | **<0.001** |  | **20.27** | **<0.001** |  | **4.14** | **0.003** |  |

Box identity is included as a random factor**.**

**Table S4** ANOVA results for effects of microplastic shape (form, fiber and bead), soil heterogeneity, patch type and their interaction on biomass of plant communities at the patch level

|  |  | Total biomass | |  | Root biomass | |  | Shoot biomass | |  |
| --- | --- | --- | --- | --- | --- | --- | --- | --- | --- | --- |
| Effect | DF | *F* | *P* |  | *F* | *P* |  | *F* | *P* |  |
| Microplastic shape (S) | 2, 3 | 7.22 | 0.071 |  | 6.80 | 0.076 |  | 0.47 | 0.629 |  |
| Soil heterogeneity (H) | 1, 58 | **12.13** | **0.001** |  | **8.60** | **0.005** |  | **5.27** | **0.025** |  |
| Patch type (P) | 1, 61 | 0.47 | 0.495 |  | **6.76** | **0.012** |  | 0.64 | 0.428 |  |
| S × H | 2, 58 | **19.36** | **<0.001** |  | **21.74** | **<0.001** |  | 2.16 | 0.124 |  |
| S × P | 2, 61 | **7.63** | **0.001** |  | **18.06** | **<0.001** |  | 1.26 | 0.292 |  |
| H × P | 1, 61 | 0.14 | 0.710 |  | **17.00** | **<0.001** |  | **4.97** | **0.030** |  |
| S × H × P | 2, 61 | **10.53** | **<0.001** |  | **36.62** | **<0.001** |  | **6.48** | **0.003** |  |

Box identity and microplastic type (EPS, PET, PP, HDPE, PLA and PA6) are included as random factors**.**

**Table S5** ANOVA results for effects of microplastic type (EPS, PET, PP, HDPE, PLA and PA6), soil heterogeneity, patch type and their interactions on shoot biomass of each plant species at the patch level

|  |  | *Elymus*  *dahuricus* | |  | *Lolium*  *perenne* | |  | *Plantago*  *asiatica* | |  | *Taraxacum*  *mongolicum* | |  | *Medicago*  *sativa* | |  | *Trifolium*  *repens* | |
| --- | --- | --- | --- | --- | --- | --- | --- | --- | --- | --- | --- | --- | --- | --- | --- | --- | --- | --- |
| Effect | DF | *F* | *P* |  | *F* | *P* |  | *F* | *P* |  | *F* | *P* |  | *F* | *P* |  | *F* | *P* |
| Microplastic type (M) | 5, 55 | **2.65** | **0.032** |  | **2.59** | **0.036** |  | 1.35 | 0.258 |  | **3.94** | **0.004** |  | **2.52** | **0.04** |  | **3.62** | **0.007** |
| Soil heterogeneity (H) | 1,55 | 0.18 | 0.675 |  | **7.24** | **0.009** |  | **18.21** | **<0.001** |  | 0.07 | 0.789 |  | 1.83 | 0.182 |  | 1.93 | 0.171 |
| Patch type (P) | 1, 55 | 0.10 | 0.756 |  | 0.38 | 0.538 |  | 0.01 | 0.940 |  | <0.01 | 0.988 |  | 2.65 | 0.109 |  | 1.54 | 0.221 |
| M × H | 5, 55 | 0.40 | 0.849 |  | 1.61 | 0.173 |  | 0.46 | 0.806 |  | 0.28 | 0.924 |  | 1.75 | 0.138 |  | **5.65** | **<0.001** |
| M × P | 5, 55 | 0.90 | 0.486 |  | 2.04 | 0.087 |  | 1.20 | 0.320 |  | 1.76 | 0.136 |  | 1.44 | 0.224 |  | 1.19 | 0.328 |
| H × P | 1, 55 | 3.88 | 0.054 |  | 0.86 | 0.357 |  | <0.01 | 0.993 |  | 0.18 | 0.673 |  | 0.03 | 0.858 |  | 0.47 | 0.495 |
| M × H × P | 5, 55 | 0.46 | 0.806 |  | 1.34 | 0.263 |  | 1.11 | 0.363 |  | 0.72 | 0.615 |  | 1.15 | 0.343 |  | 0.96 | 0.453 |

Box identity is included as a random factor**.**

**Fig. S1** Shoot mass of (A) *Elymus dahuricus*, (B) *Lolium perenne*, (C) *Medicago sativa*, (D) *Trifolium repens*, (E) *Plantago asiatica* and (F) *Taraxacum mongolicum* in the patches the high- and low-quality soil patches in the heterogeneous treatment and the imagined high- and low-quality patches in in the homogeneous treatment for each of the six types of microplastics. Bars and vertical lines are mean and SE. Symbols (^*^ *P* < 0.05) indicate significant differences between the two types of patches.
